# Drug Release Profiles of Mitomycin C Encapsulated Quantum Dots–Chitosan Nanocarrier System for the Possible Treatment of Non-Muscle Invasive Bladder Cancer

**DOI:** 10.3390/pharmaceutics13091379

**Published:** 2021-08-31

**Authors:** Fariza Aina Abd Manan, Nor Azah Yusof, Jaafar Abdullah, Faruq Mohammad, Armania Nurdin, Latifah Saiful Yazan, Sachin K. Khiste, Hamad A. Al-Lohedan

**Affiliations:** 1 Institute of Advanced Technology, Universiti Putra Malaysia, Serdang 43400, Selangor, Malaysia; gs56709@student.upm.edu.my (F.A.A.M.); jafar@upm.edu.my (J.A.); 2 Department of Chemistry, Faculty of Science, Universiti Putra Malaysia, Serdang 43400, Selangor, Malaysia; 3 Department of Chemistry, College of Science, King Saud University, P.O. Box 2455, Riyadh 11451, Saudi Arabia; hlohedan@ksu.edu.sa; 4 Department of Biomedical Sciences, Faculty of Medicine and Health Sciences, Universiti Putra Malaysia, Serdang 43400, Selangor, Malaysia; armania@upm.edu.my (A.N.); latifahsy@upm.edu.my (L.S.Y.); 5 Department of Medicine, Harvard Medical School, Boston, MA 02115, USA; khiste@bwh.harvard.edu

**Keywords:** chitosan nanocarrier, Mn:ZnS quantum dots, drug delivery systems, mitomycin C delivery, cancer cell therapy

## Abstract

Nanotechnology-based drug delivery systems are an emerging technology for the targeted delivery of chemotherapeutic agents in cancer therapy with low/no toxicity to the non-cancer cells. With that view, the present work reports the synthesis, characterization, and testing of Mn:ZnS quantum dots (QDs) conjugated chitosan (CS)-based nanocarrier system encapsulated with Mitomycin C (MMC) drug. This fabricated nanocarrier, MMC@CS-Mn:ZnS, has been tested thoroughly for the drug loading capacity, drug encapsulation efficiency, and release properties at a fixed wavelength (358 nm) using a UV–Vis spectrophotometer. Followed by the physicochemical characterization, the cumulative drug release profiling data of MMC@CS-Mn:ZnS nanocarrier (at pH of 6.5, 6.8, 7.2, and 7.5) were investigated to have the highest release of 56.48% at pH 6.8, followed by 50.22%, 30.88%, and 10.75% at pH 7.2, 6.5, and 7.5, respectively. Additionally, the drug release studies were fitted to five different pharmacokinetic models including pesudo-first-order, pseudo-second-order, Higuchi, Hixson–Crowell, and Korsmeyers–Peppas models. From the analysis, the cumulative MMC release suits the Higuchi model well, revealing the diffusion-controlled mechanism involving the correlation of cumulative drug release proportional to the function square root of time at equilibrium, with the correlation coefficient values (R^2^) of 0.9849, 0.9604, 0.9783, and 0.7989 for drug release at pH 6.5, 6.8, 7.2, and 7.5, respectively. Based on the overall results analysis, the formulated nanocarrier system of MMC synergistically envisages the efficient delivery of chemotherapeutic agents to the target cancerous sites, able to sustain it for a longer time, etc. Consequently, the developed nanocarrier system has the capacity to improve the drug loading efficacy in combating the reoccurrence and progression of cancer in non-muscle invasive bladder diseases.

## 1. Introduction

Recent years have witnessed the unprecedented growth of research and applications in the field of nanotechnology-based drug delivery systems (DDS), especially for cancer diagnostic and treatments, as cancer has been garnering tremendous interest because of its severity to cause death worldwide and threatening public health [1]. Since the carcinogenesis process is tedious, it has limitation on treatment regiments, and requires more rigorous and comprehensive therapeutic plans. Although numerous treatment modalities, for example immunotherapy, phototherapy, gene therapy and hormone therapy, are emerging, nonetheless, the gold standard for cancer treatment goes to surgical intervention and chemotherapy [2]. It is noteworthy to mention that conventional chemotherapy is considered as a non-specific treatment that can simultaneously kill healthy cells and leads to systemic toxicity to the patients [3]. Thus, the quest for innovative technologies becomes an urgent necessity.

In that view, innovative technologies with diverse nanomaterials have been implemented as nanocarrier systems to encapsulate many kinds of drugs due to their special characteristics such as small particle size, high surface area, surface charges, etc. [4]. In contrast, the conventional methods for the administration of several drugs by chemotherapeutic approaches exhibit excellent curative effects; however, they suffered from massive downsides that may lead to adverse side effects on healthy tissues prior to their poor aqueous solubility, inadequate drug concentration at the lesion site, non-specific biodistribution, intolerable cytotoxicity and the development of multiple drug resistance, severely limit the therapeutic efficacy, and cause undesirable side effects [5]. Overall, to prevail these shortcomings, the DDS has been implemented with high specificity, good biodistribution, prolonged systemic circulation, low toxicity, and invasive molecular imaging for targeted and controlled DDS [6]. Besides, the DDS has improved the biocompatibility of drug with the cells and tissues, increased the intracellular uptake, retained the stability of drugs, and improved the ability of drugs to be delivered to the target specific cells or tissues while sparing the normal cells [7].

In this regard, chitosan (CS)-based nanocarrier systems find multifaceted applications in DDS because of its biocompatility, biodegradability, non-toxicity, and antimicrobial properties. In brief, chitosan is a biodegradable polymer of polysaccharide consisting of alternate repeating units of (1→4) linked *N*-acetyl glucosamine and glucosamine units by glycosidic links, derived from partial deacetylation of chitin [8,9,10,11]. The CS molecules can be incorporated as part of films, microspheres, nanospheres, and nanoparticles (NPs), and are eligible to be conjugated with other nanomaterials for broad applications of biocatalysis, biomedicine, and pharmaceutical sectors. Basically, the CS molecules can be transformed into nanocarriers with definite particles sizes and particle surface charges by altering its molecular weight and degree of acetylation. The CS-based nanocarrier encapsulated drugs are found to be very effective for theranostics applications because of the hemocompatibility, antibacterial activity, particle size and shape, surface charge and morphology accompanied by the presence of targeting ligands strongly affecting the effectiveness of cell targeting, internalization, and anti-tumor action of cancer therapeutics [12,13].

On this note, the potential of CS is deemed to be applied in DDS by conjugating with metal and polymer-based nanomaterials. Of many, some of the CS nanocarriers are containing iron oxide (Fe_3_O_4_), silicon, quantum dots (QDs), polyethylene glycol (PEG), polylactic acid glycolic acid (PLGA), etc., used for the delivery of anticancer drugs [14,15,16]. Such an incorporation of both metallic particles and anticancer drugs within a single nanoparticulate polymer system in the form of nanocarrier has evoked fascinating outcomes and substantial progress in DDS for cancer diagnosis and treatment. In general, the QDs are semiconductor nanocrystals (NC) made up of atoms or elements from group II–VI or from group III–V with small particles size, which are smaller than exciton Bohr radius. The QDs exhibit unique optical and electronic properties prior to their quantum confinement effect. Most of the metal and semiconductor NPs with particle sizes ranging from 2 to 6 nm have received mounting interest because of their unique size-dependent properties, as well as their dimension that mimics the biological macromolecules [17]. In recent years, QDs have been widely employed as imaging agent rather than other fluorescent nanomaterials and dyes. Briefly, QDs have drawn much attention because of the advantages of multiplex emission with single light excitation with minimal overlap [18]. Additionally, QDs exhibit excellent optoelectronic properties such as high quantum yield, high photostability, broad excitation wavelength-dependent optical emission [19], broad and tunable absorption spectrum extending from the ultraviolet (UV) to near-infrared emission (>650 nm), size-tunable light emission broad absorption spectrum, massive stoke shift and resistance to photobleaching [18,20,21].

These properties of QDs allow for the simultaneous application in bioimaging cancerous tissues and also act as photosensitizers in photodynamic therapy [22]. Hence, the integration of QDs as fluorescence probe in various applications such as photocatalysis, bioimaging, biosensing, biomedicine, and DDS has been boosted instead of the conventional organic luminophores due to their intrinsic ability to resist photobleaching [23,24].

The conjugation of CS and its derivatives with numerous QDs for biomedical application has been widely explored. According to the literature, the conjugation of CS with QDs shows no significant toxicity when examined in an in vivo evaluation in mice [25]. Typically, the surface coating of the inorganic QDs is crucial to retain the stability of colloidal NPs, prevent the degradation of QDs, and reduce the toxicity of inorganic metal ions [20]. In recent years, the ZnS QDs has dominated broad applications due to their optical properties, elevated refractive index, wide band gap, and good luminescence. However, pure ZnS has poor optoelectronic properties and low quantum efficiency and so, the doping of ZnS with various transition metal atoms has been explored to diversify the optical and structural properties in the ZnS host lattice. There are numerous transition metal atoms that have been used as doping elements for ZnS, but Mn has been widely used because of the properties of excellent luminescence, close ionic radius and ionic charge that can imitate Zn^2+^, good microstructure, electrical and optical properties, enhanced thermal and photostabilities [26].

In the present report, the novelty of our studies lies in the fabrication of a stable and biocompatible CS-based nanocarrier conjugated system having Mn:ZnS QDs (CS-Mn:ZnS) to improve the bioavailability of mitomycin c (MMC, an anticancer drug) for active targeted cancer cell therapy. To the best of the authors knowledge, the present work sought to explore MMC encapsulation onto CS-Mn:ZnS, which has not yet been reported previously. In addition, the architecture of drug nanocarriers for MMC is tremendously difficult because MMC is classified as a water soluble drug that faced great challenges in cell penetration and internalization prior to the lipophilic nature of cell membranes. We present the first report on the formulation for MMC, that has been successfully encapsulated onto CS-Mn:ZnS nanocomposite matrix, even though MMC is a water-soluble drug that suffers from the limitations of rapid or burst release in aqueous solutions. As a result, the MMC@CS-Mn:ZnS nanocarriers convey an excellent internalization and are engulfed into the targeted cancer cell with high sustainability and extend the MMC efficiency for targeted non-muscle invasive bladder cancer therapy. The excellent results of this formulation may provide constructive hints for further developments in this research area of DDS and theranostics.

## 2. Materials and Methods

### 2.1. Materials

Mitomycin C (MMC; C_15_H_18_N_4_O_5_, Mw = 334.33 g·mol^−1^) was purchased from Tocris Bioscience (Bristol, UK). Chitosan (CS; medium molecular weight, 190,000–310,000 degree of acetylation), Tween-20 (C_26_H_50_O_10_, Mw = 522.7 g·mol^−1^), and sodium tripolyphosphate, TPP (Na_5_O_10_P_3_, Mw = 367.86 g·mol^−1^) were purchased from Sigma-Aldrich (St. Louis, MO, USA). Zinc acetate dihydrate (Zn(CH_3_COO)_2_·2H_2_O) (Mw = 183.48 g·mol^−1^, 99.5%) sodium sulphide (Na_2_S⋅xH_2_O (x = 7–9), Mw = 240.18 g·mol^−1^, yellow flakes), manganese sulfate monohydrate (MnSO_4_⋅H_2_O, Mw = 169.02 g·mol^−1^, 99%), and sodium tripolyphosphate (Na_5_O_10_P_3_, Mw = 367.86 g·mol^−1^, 59%) were procured from R&M Marketing (Essex, UK). Hydrochloric acid (HCl, 36.458 g·mol^−1^, 37%) was purchased from Friendemann Schmidt (Parkwood, Australia). Other reagents were of analytical grade and used without any further purification. All aqueous solutions were prepared using ultrapure water of resistivity (18.2 MΩ·cm), purified using Thermo Scientific water purification system.

### 2.2. Preparation of MMC@CS-Mn:ZnS Nanocarriers

The synthesis of fluorescence probe, Mn:ZnS QDs was carried out by modifying the previously described method [27]. Initially, 0.15 M (0.076 g, for 3 mL) of MnSO_4_·H_2_O, 0.10 M (0.367 g, for 20 mL) of Zn(CH_3_COO)_2_·2H_2_O, and 0.1 M (0.480 g, for 20 mL) of Na_2_S⋅xH_2_O were prepared in deionized water, separately. Then, 3 mL of 0.15 M MnSO_4_·H_2_O was added dropwise into 20 mL of 0.10 M of Zn(CH_3_COO)_2_·2H_2_O, in ultrasonic bath that operates at a frequency of 40 kHz. The solution was de-aerated using N_2_ gas for 15 min to remove unwanted dissolved gases that may disrupt the synthesis process, followed by the injection of 20 mL of 0.10 M of Na_2_S⋅xH_2_O drop by drop under constant magnetic stirring at 200 rpm, with continuous nitrogen purging. The presence of fluorescence emission can be observed under handheld UV lamp. The resulting precursor of Mn-doped ZnS (Mn:ZnS) was exposed to microwave irradiation at 1000 W, using sealed Teflon reaction vessels at 120 °C for 60 s to accelerate processing time and improve the purity of prepared nanostructures [28]. Then, the suspension was further exposed to UV irradiation for 20 min.

In the next stage, MMC was loaded onto CS-Mn:ZnS nanocarrier and for that, the ionic gelation method with slight modification was employed [28]. Briefly, CS solution (5 mg/mL) was prepared by dissolving 5 mg of CS powder in 1 mL of 1.0% (*v*/*v*) acetic acid solution. Then, 2.5 µL Mn:ZnS was added dropwise into 250 µL of CS solution under constant stirring. In a different tube, 1 mg/mL of MMC was prepared in deionized water separately. Next, the 250 µL of CS solution and 250 µL of MMC with ratio of 1:1 (*v*/*v*) were mixed under sonication until a homogeneous solution was formed. Then, 2% (*v*/*v*) Tween-20 was dispersed in deionized water, added to prevent particle aggregation with the volume ratio of 1:100 (*v*/*v*) for Tween-20 to the CS solution. In this study, Tween-20 acts as a stabilizing, capping agent that forms interaction with water molecules through its hydrophilic domains, and hence accelerates the interaction of NPs with the aqueous medium. It is noteworthy to mention that Tween-20 is a safe addition to biomedical NPs formulations [29].

Despite its natural properties, Tween-20 provides stability to NPs [30]; the reason it was added at this stage was because the formation of NPs takes place at this step, where CS NPs were formed spontaneously upon the addition of 100 µL TPP (10 mg/mL) drop-by-drop using a micropipette under continuous magnetic stirring at 200 rpm. The final TPP-to-CS ratio achieved was 1.0:2.5 (*v*/*v*). The mixture was then centrifuged at 12,000 rpm for 10 min against deionized water for three times and the supernatant was discarded. Finally, the MMC@CS-Mn:ZnS nanocarrier pellet was then freeze-dried overnight before further analysis.

### 2.3. Reaction Yield, Drug Loading, and Encapsulation Efficiency

The reaction yield obtained for MMC@CS-Mn:ZnS nanocarriers was evaluated using Equation (1) [28].

Reaction yield (RY) = (total mass of nanocarriers produced (mg))/(mass of chitosan (mg) + mass of Mn:ZnS (mg) + mass of MMC (mg)) × 100(1)

The loading capacity (LC) and encapsulation efficiency (EE) of MMC was evaluated using Nanodrop Spectrofluorometer at wavelength of 358 nm. Typically, 5.0 mg of resulting nanocarriers was dissolved against the mixture of methanol and 0.5% HCl (*v/v*) under sonication until a clear solution was observed, indicating that the nanocarriers were totally dissolved and hence, release 100% of entrapped MMC drug inside the nanocarriers. Finally, LC and EE were successfully calculated using Equations (2) and (3) as follows:

Loading capacity, LC (%) = [encapsulated MMC in nanocarriers (mg)]/[Mass of nanocarriers used (mg)] × 100(2)

Encapsulation efficiency, EE (%) = [encapsulated MMC in nanocarriers (mg)]/[Initial mass of MMC in the system (mg)] × 100(3)

### 2.4. MMC Drug Release Studies

The MMC release profile from Mn:ZnS nanocarriers was quantified using Multiskan GO Microplate Spectrophotometer at wavelength of 358 nm. Initially, 10.0 mg of the synthesized nanocarriers was dispersed into 10 mL of phosphate-buffered saline (PBS) solution with pH 6.5, pH 6.8, pH 7.2 and pH 7.5 under constant stirring. The pH for PBS was chosen to imitate the medium in human urine. At predetermined intervals of time, 1 mL of the solution was discarded by centrifugation and replaced with the same amount of fresh medium.

### 2.5. Characterization of MMC@CS-Mn:ZnS Nanocarriers

The UV–Vis and PL (photoluminescence) analyses were carried out to study the photophysical properties of the ZnS and Mn:ZnS nanostructures. The Fourier transform infrared spectra (FTIR), powder X-ray diffraction (XRD) were used to characterize the functionality and crystal structure of the synthesized materials (respectively). The FTIR spectra of the samples were obtained at ambient temperature using attenuated total reflectance (ATR) technique in the wavenumber range of 500–4000 cm^−1^ using a series 100 Perkin Elmer FTIR 1650 spectrophotometer (Perkin Elmer, Waltham, MA, USA). The phase and purity of NPs was acquired using an X-ray diffractometer (Rigaku SmartLab, Tokyo, Japan) operating at a scanning rate of 1°/min. The diffraction spectra were recorded at the diffraction angle, 2θ from 20° to 70° at room temperature. The hydrodynamic particle size was determined by dynamic light scattering (DLS) studies using a particle size analyzer (Nano Series Nano-ZS, Malvern Panalytical Ltd., Malvern, UK). The internal morphology and particle size diameter were studied using a high-resolution transmission electron microscope (HRTEM), FEI Tecnai G2 F20 S-TWIN (Hillsboro, OR, USA). The surface morphology studies were conducted using Field-emission scanning electron microscopy (FESEM) attached with EDX (JSM-7500F JEOL, Tokyo, Japan). The amount of drug loading and release was measured using Multiskan GO Microplate Spectrophotometer (Thermo Fischer Scientific, Waltham, MA, USA) at a wavelength of 358 nm.

### 2.6. Statistical Analysis

Data are presented as the mean ± standard deviation and the statistical difference of parameters was analyzed using ANOVA with Tukey’s and Bonferroni’s model (where applicable) for p test with (*p* < 0.05). A *p*-value of less than 0.05 was considered as statistically significant. The full width at half maximum (FWHM) of XRD diffraction peaks for all NPs were acquired using Gauss and Lorentz fitting function. All statistical analyses were performed using Origin 8 Software (Microcal Software, Inc., Northampton, MA, USA).

## 3. Results and Discussion

### 3.1. Characterization of ZnS and Mn:ZnS QDs

#### 3.1.1. UV–Vis Spectroscopy

**Figure 1:**
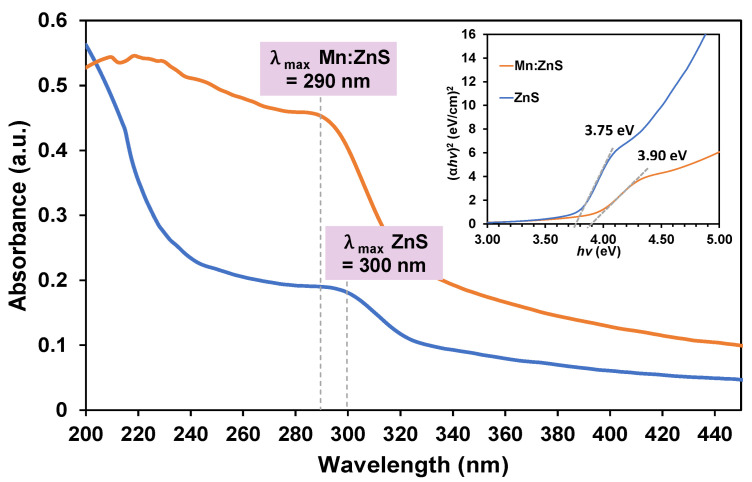
Comparison of UV–Vis spectra for ZnS and Mn:ZnS QDs (inset showing the Tauc’s plot for the same two samples).

Figure 1 illustrates the UV–Vis spectra of ZnS and Mn:ZnS NPs at room temperature in the wavelength range of 200–600 nm and from the graph, it is clearly observed for an enhancement in the absorption intensity of Mn:ZnS spectra. This can be attributed to the effect of quantization and alteration of the defect states within crystalline lattice structure of ZnS with appropriate substitution of Mn^2+^ to Zn^2+^ that possesses different sizes (as reported previously) [26,30]. In addition, the absorption edges were observed at 300 and 290 nm for ZnS and Mn:ZnS, respectively, with a tail extending into the visible region, which indicates that the NPs are exhibiting good crystallinity and low defect density near the band edge [12]. Additionally, there is a hypsochromic blue shift (~10 nm) in the shoulder peak from red region of 300 nm to blue region of 290 nm in Mn:ZnS absorption edge. Such an observation may be accredited to the formation of smaller particles than that of bulk ZnS and the quantum confinement effect of Mn:ZnS NPs, consistent with the previous reports on semiconducting ZnS QDs [22,31,32]. The band gap of chemically synthesized ZnS and Mn:ZnS NPs were obtained from the UV absorption data using the following Equation (4):

α*hυ* = A (*hυ* − *E_g_*)^1/2^ (4)

where α is the absorption coefficient, A is a proportionality constant, *hv* is the photon energy and *E_g_* is the band gap energy of the material [31]. The *hv* was derived from *hv* = hc/λ, where h is the Planck constant (4.136 × 10^−15^ eV), c is the velocity of light in vacuum (2.997 × 10^17^ nm/s), and λ is the wavelength (nm) [33]. Further, Figure 1 (inset) shows the plot of (α*hv*)^2^ versus *hv* for both ZnS and Mn:ZnS. The band gap value can be simply obtained by extrapolating the straight portion of (α*hv*)^2^ versus *hv* with the value of α = 0. The values for band gaps were obtained from the extrapolated Tauc’s plot at 3.75 and 3.90 eV for ZnS and Mn:ZnS, respectively.

The particle sizes of both ZnS and Mn:ZnS were also calculated from the UV–Vis absorption spectra using Equation (5):

(5) $$D=\frac{0.32-2.9\;{\left(E_{g}-3.49\right)}^{\frac{1}{2}}}{3.50-E_{g}}$$

where, *E_g_* is the band gap (in eV) and *D* is the diameter of nanocrystallites in nm [34]. By using the above equation, the diameters of particles were obtained to be 4.63 and 3.84 nm for ZnS and Mn:ZnS, respectively. This data will be further supported by transmission electron microscopy (TEM) data and will be further discussed in Section 3.1.3.

#### 3.1.2. PL Spectroscopy

**Figure 2:**
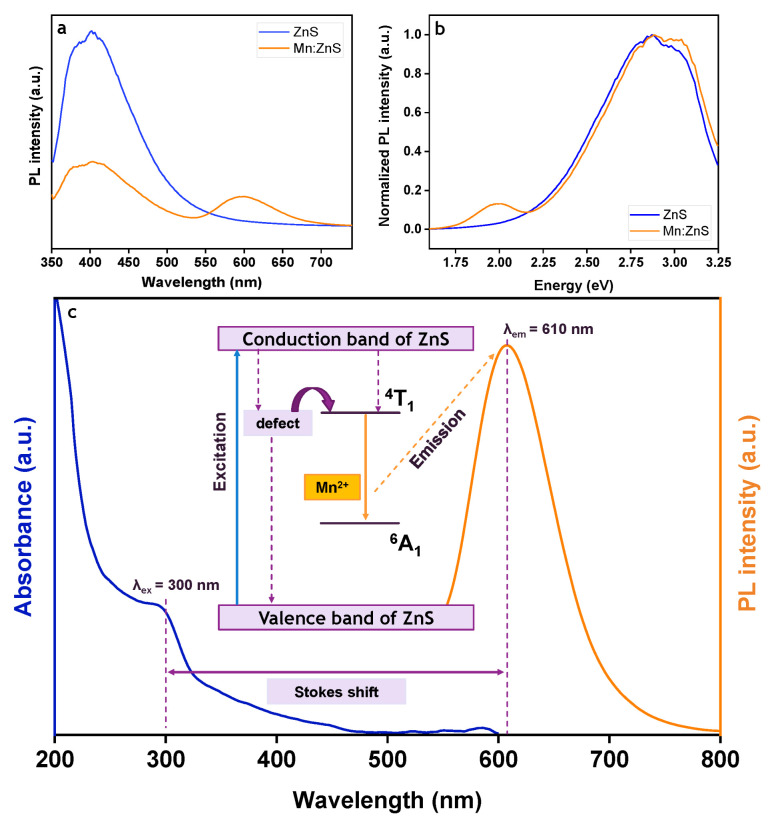
Comparison of (**a**) PL spectra, (**b**) normalized PL spectra, and (**c**) schematic representation for electron transition among ZnS and Mn:ZnS nanostructures.

The room temperature PL spectra for ZnS and Mn:ZnS are compared in Figure 2a where we observed the PL peak for ZnS at ~400 nm. However, for the Mn:ZnS, two symmetrical peaks were recorded at ~400 nm (violet-blue region) and ~600 nm (red region). The presence of new emission band at ~600 validates that the Mn^2+^ successfully occupied the Zn^2+^ tetrahedral cation sites with Td symmetry in ZnS lattice. It is worth mentioning that the ZnS exhibits optically active defect states in the band gap due to available S and Zn vacancies (V_S_ and V_Zn_) emitting at 440 and 520 nm, respectively [35].

Figure 2b illustrates the normalized PL intensity of ZnS with emission energy of ~3.00 eV. The PL peak for Mn:ZnS was prominently hypsochromic shifted to the blue region and might be due to the cation exchange [36]. In addition, the shifting of PL intensity from ~400 to 410 nm, in which towards the longer wavelength suggests that the transition of electrons from conduction band to the electron trap centers, the least quantum energy was transmitted to the lattice or Mn states. Hence, the reduction or quenching in Mn emission energy was observed.

The blue fluorescence emission in pure ZnS was diminished after Mn was successfully doped onto the ZnS lattice structure. This phenomenon is due to the mixing of s-p electrons of the host ZnS with the d-electron of Mn^2+^ and leads to the forbidden partial transition of electron from ^4^T_1_ to ^6^A_1_ state as shown in Figure 2c, emitting orange fluorescence [37,38]. The reduction of PL peak intensity can be attributed to (i) the weak Mn-Mn interaction (due to low doping concentration) with s-p electrons in nanophase materials, and (ii) strong hybridization of Zn^2+^ and Mn^2+^ with crystal field splitting of Mn^2+^ [39]. It should be noted that Stokes shifts were calculated from both absorbance and emission maxima wavelengths. In this work, the Stokes shift is inevitably large with the value of 310 nm, in which large and tunable Stokes shift is crucial to achieve precise imaging, with unlimited application in molecular imaging [40].

#### 3.1.3. HRTEM Analysis

**Figure 3:**
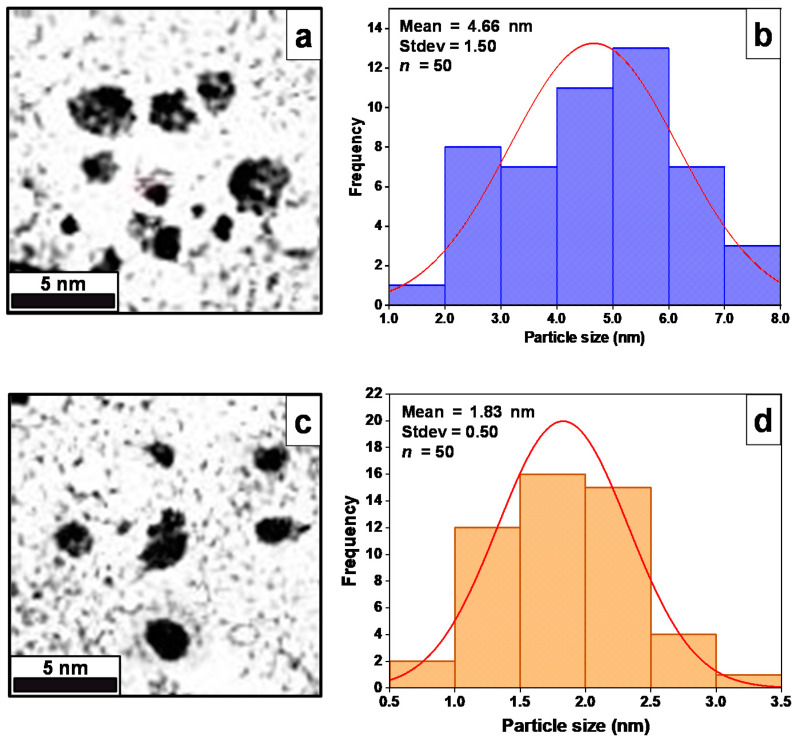
HRTEM images of (**a**) ZnS and (**c**) Mn:ZnS QDs; corresponding particle size distributions for (**b**) ZnS and (**d**) Mn:ZnS.

Both ZnS and Mn:ZnS samples were diluted with deionized water with the ratio factor of 1:10 (*v*/*v*) for (ZnS/Mn:ZnS):deionized water. The mixture was deposited onto the Cu grids covered with carbon film (200 mesh) using a disposable dropper and a standard filter paper was used to absorb the excess solvent according to Ribeiro et al. [23]. Figure 3a shows the monodispersed spherical shape ZnS particles with average particle size of 4.66 nm [41]. Similarly, the HRTEM micrograph for Mn:ZnS exhibit spherical NPs with average particle size of 1.83 nm, as shown in (**c**). The reduction in particle size is in agreement with the results acquired from structural analysis [42]. The particle size distributions for ZnS (**b**) and Mn:ZnS (**d**) were evaluated using ImageJ software and plotted using Origin 8 software.

#### 3.1.4. FTIR Spectroscopy

**Figure 4:**
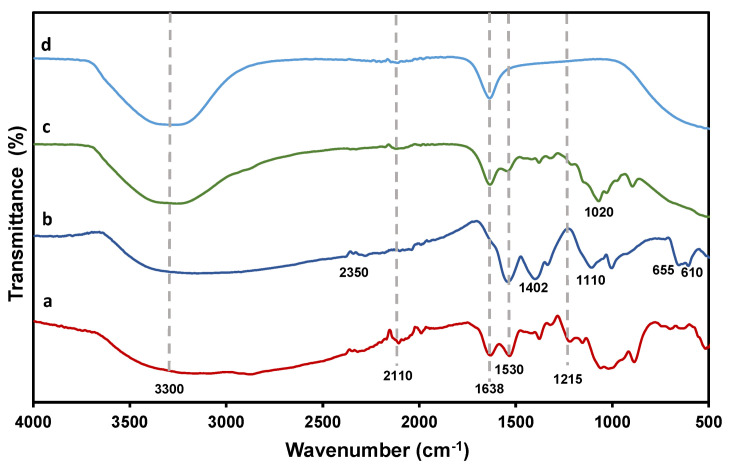
FTIR spectral comparison of (**a**) CS, (**b**) Mn:ZnS, (**c**) MMC@CS-Mn:ZnS, and (**d**) MMC samples.

FTIR analysis attempted to characterize the conjugation interaction of single NPs by comparing the spectra of (**a**) CS, (**b**) Mn:ZnS, (**c**) MMC@CS-Mn:ZnS, and (**d**) MMC as illustrated in Figure 4. For CS (**a**) there is broad band at ~3300 cm^−1^ and prior to the C-H bond and NH_2_ stretching as mentioned before by previous work [23] and the peak becomes more intense in MMC@CS-Mn:ZnS probably due to the ingestion of MMC onto the nanocomposite. There is a weak peak at ~2350 cm^−1^ in Mn:ZnS spectrum due to the Zn–S microstructure vibration, but disappeared in the spectra of CS-Mn:ZnS and can be attributed to the interaction between the Zn–S and the carboxylate groups of CS to form the nanocomposite [27]. Next, the absorption peaks present at ~2110 cm^−1^ are mainly assignable to CO stretching vibrations in CS NPs. Meanwhile, the spectral band at ~1638 cm^−1^ corresponds to the stretching vibration of C=O and amide group in CS and MMC [43]. In MMC@CS-Mn:ZnS, the peak reveals the integration of MMC and CS onto the nanocomposite. Additionally, the COO- symmetric and asymmetric stretching can be seen at the spectral band of ~1530 cm^−1^ in all spectra and an additional peak at 1402 cm^−1^ in Mn:ZnS. The peak at 1402 is disappearing in MMC@CS-Mn:ZnS sample and might be due to the formation of nanocomposites that involve the binding of carboxyl group (COO–) of Mn:ZnS with the amino group (–NH_2_) of CS [44], as the amine group of CS supports electrostatic interaction to produce a stable mixture [45].

The absorption peaks at 1110 cm^−1^ apparently in Mn:ZnS correspond to the symmetric stretching vibration of Zn–S–Mn bond [27]. Hasheminejad et al. [9] reported the observation of absorption peak with slight shifting probably due to the overlapping of C–O–C glucose ring or C–O stretching vibrations of peak at ~1030 cm^−1^ in CS sample. There is a ZnS band and symmetric bending due to Zn–S or Mn–S vibrations for Mn:ZnS at ~655 and ~610 cm^−1^, thus strongly verifying the conjugation of Mn:ZnS into the host, CS NPs [27]. All the absorption peaks are summarized in Table 1.

#### 3.1.5. XRD Analysis

**Figure 5:**
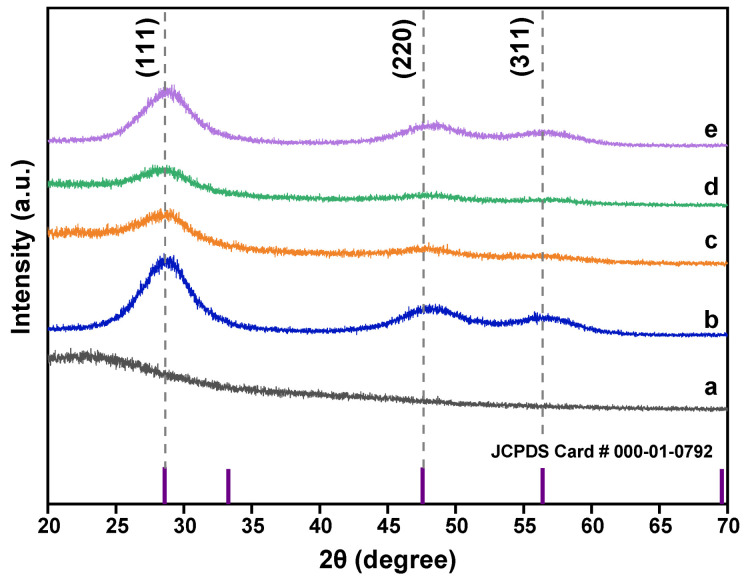
Comparison of XRD patterns for (**a**) CS NPs, (**b**) ZnS, (**c**) Mn:ZnS, (**d**) CS-Mn:ZnS, and (**e**) MMC@CS-Mn:ZnS.

The XRD patterns of all prepared samples were obtained to characterize their crystalline nature and attest their phase purity (Figure 5). From the figure, we observed the diffraction patterns for the ZnS sample at 2θ = 28.40°, 48.40°, and 55.50° (Figure 5b) and for the Mn-doped ZnS (Mn:ZnS) at 28.70°, 48.70°, and 56.40° (Figure 5c). For the comparison of both diffraction peaks, a shift in the ZnS diffraction peak slightly to the higher angle was observed due to the doping of Mn^2+^ into ZnS crystal lattice. This is prior to the lattice contraction after the substitution of Mn^2+^ into Zn^2+^ sites as Mn^2+^ exhibit larger atomic radius compared to Zn^2+^ ions. The broadening of diffraction peaks of Mn:ZnS after doping process designate the formation of nanostuctured particles. Additionally, the observation of a reduction in intensity of Mn:ZnS diffraction peaks validates that the size and crystalline nature of pure ZnS was deteriorated. In the recorded XRD pattern, the impurity peaks are absent, revealing that the doped Mn ions are getting properly substituted into ZnS lattice without much alteration to the basic structure of ZnS and with minimum shrinkage and distortion to the crystal lattice [46]. The observed patterns can be correlated to the reflection planes of (111), (220), and (311) for ZnS and Mn:ZnS QDs in the matrix; the hkl diffraction plane suggests cubic zinc blende phase in accordance with Joint Committee on Powder Diffraction Standard (JCPDS Card No.000-01-0792) [31]. The hkl diffraction planes exhibit space group of F-43m with the space group number 216 matching with the ZnS nanoctructure, as reported in the literature [31,42]. Further, the observation of diffraction reflections for the pure CS NPs (Figure 5a), CS-Mn:ZnS (Figure 5d), and MMC@CS-Mn:ZnS (Figure 5e) without any impurity peak suggest the purity of polymeric nanostructure and accuracy of the product [47].

The average crystallite sizes, (*D*) of synthesized NPs were calculated using the Debye-Scherrer’s Equation (6):

(6) $$D=\frac{k\lambda }{\beta \cos\theta }$$

where, *D* is the crystallite size, *k* the shape factor (assumed to be 0.89 for spherical shape as confirmed by HRTEM image), *λ* is the wavelength of incident X-ray radiation of CuKα (0.154 nm), *β* is the full width at half maximum (FWHM) of plane, and *θ* is the Bragg’s diffraction angle [31]. In this regard, the highest intensity diffraction peak with (111) crystal plane was selected. The variation in peak position (2θ), FWHM, d-value, and average crystalline size along (111) plane, dislocation (δ), and microstrain (ε) for the ZnS, Mn:ZnS, CS-MN:ZnS, and MMC@CS-Mn:ZnS. The increase of FWHM was observed as the crystallite size decreased.

The broadening of diffraction peaks is mainly due to two factors, (i) size in the quantum regime and (ii) strain induced in the nanostructures. Other than that, the peak broadening might be due to a linear combination of the nanocrystalline nature and local strain in the nanostructure due to the defects [46]. The strain (*ε*) prior to crystal imperfection and distortion was calculated using Stokes–Wilson Equation (7) [48]:

(7) $$strain\;\left(\varepsilon \right)=\frac{\beta \;\cos\theta }{4}$$

The number of dislocations presenting in the unit area of synthesized samples were calculated using Equation (8):

(8) $$\delta =\frac{1}{D^{2}}$$

The lattice constant, *a* for the cubic structure was determined via [111] orientation using the following Equation (9):

(9) $$a^{2}=d_{hkl}{}^{2}\left(h^{2}+k^{2}+l^{2}\right)\;$$

where *h*, *k*, *l* are the Miller indices and *d_hkl_* is the interplanar space calculated from Bragg’s equation: 2*d*Sin*θ* = *n*λ. All data obtained from the diffraction analysis are tabulated in Table 2.

#### 3.1.6. Morphology and Particle Size Distribution

**Figure 6:**
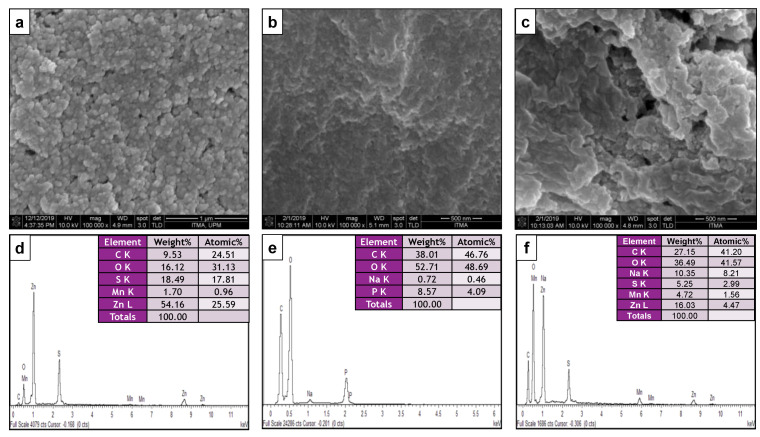
FESEM images for (**a**) Mn:ZnS; (**b**) CS, and (**c**) CS-Mn:ZnS; EDX spectra for (**d**) Mn:ZnS, (**e**) CS, and (**f**) CS-Mn:ZnS nanostructure.

The surface morphological characterizations of as-synthesized composite acquired from FESEM attached to energy dispersive X-ray spectroscopy (FESEM-EDX) is provided in Figure 6a–c. From the images, the micrographs obtained for Mn:ZnS (Figure 6a) show a smooth surface with spherical shape of the particles. On the other hand, the pure CS and CS-Mn:ZnS nanocomposite (Figure 6b,c) incurred agglomeration, which can be clearly seen in the FESEM images. The EDX elemental analysis for Mn:ZnS (Figure 6d) shows the presence of Zn, S, and Mn (1.70 wt%) elements, confirming that Mn has been successfully doped into ZnS nanostructure.

EDX was employed to investigate the elemental analysis, by determining the elemental composition of individual points or to map out the lateral distribution of elements from the imaged area. Other than that, the EDX has been widely used to study the compositional information on quasi-bulk specimens (low SEM magnification, high accelerating voltage) or on specific particles, morphologies, or isolated areas on filters or within deposits [49].

For the pure CS NPs (Figure 6e), the EDX analysis exhibits the composition of carbon and oxygen, with the presence of Na (0.72%) and P (8.57 wt%), validating the presence of TPP in CS [50]. For the CS-Mn:ZnS nanocomposite (Figure 5f), the combination of composition from Mn:ZnS and CS is observed, thereby revealing the successful conjugation of CS with that of Mn:ZnS QDs.

Furthermore, the hydrodynamic size and polydispersity index (PDI) for the formulated nanocarriers measured by the DLS analysis are shown in Table 3. From the data, the particle size resembles the size of NPs, while PDI resembles homogeneity of particles distribution; lower PDI samples are made up of more uniform particles size and, therefore, they are more monodispersed. Meanwhile, zeta potential measures the surface charge of NPs that gets developed at the particle–liquid interface [50]. In this study, the drug nanocarriers were successfully synthesized by ionic gelation method using biodegradable CS crosslinked with TPP. The TPP was chosen rather than other crosslinkers prior to its low toxicity and no possibility of causing antigenicity. Briefly, the multivalent anions (–P_3_O_10_^5−^) interact with (–NH_3_^+^) (after CS has been protonated under acid condition using acetic acid) by inter- and intramolecular cross-linking interaction, serving as the basis of ionic gelation process for the formation of CS NPs [51,52]. The size, PDI, and zeta potential of all synthesized particles provided in Table 3 indicate that the MMC@CS-Mn:ZnS has the particle size of 175 nm and PDI value of 0.448. In terms of particles size, we observed an increase for the MMC@CS-Mn:ZnS as compared to naked CS-Mn:ZnS (before drug loading), indicating that the incorporation of drug molecules into the nanoparticulate structure have resulted in an increased particle size, and at the same time confirming the efficient loading of MMC into the CS matrices [53,54]. Generally, zeta potential (ZP) is an analytical technique to quantify the surface charge of NPs in colloidal solutions. As the surface charged particles attract a thin layer of opposite charge, they bind to it, forming a thin liquid layer called a Stern layer. Next, the diffusion of particles in the aqueous medium will encourage ion interaction in which those loosely associated at the outer diffuse layer result in the formation of double layer. The electrical potential of the double layer known as ZP, typically lies in the range of −100 to +100 mV [55]. Additionally, the ZP can be used to determine the degree of repulsion between the charged particles in the dispersion. The NPs with high ZP or high charged particles (positive ZP) will tend to resist aggregation prior to the electric repulsion. Meanwhile, the NPs with low ZP (negative ZP) will attain attraction rather than repulsion, which leads to the formation of coagulated particles [56]. The ZP values ranging from 20 to 40 mV are likely to be the optimum condition to confer good stabilization of a nanodispersion and less prone to form aggregations, even though the particles sizes increase [57,58].

As for the ZP, the MMC@CS-Mn:ZnS exhibit surface charge with the value of +33.20 ± 0.38 mV, which is significantly no different from the pure CS (+32.60 ± 0.29 mV) and CS-Mn:ZnS (+32.70 ± 0.46 mV), suggesting that the MMC drug did not undergo conjugation with the CS matrix, but was successfully encapsulated by physical bonds [51]. The surface charge of all CS-based NPs is around +30 mV, which is in a good agreement with the previous report and in addition, the observation of assimilated positive charges can be due to the presence of positively charged amino groups (−NH_3_^+^) in CS’s polymeric chain [50]. Substantially, the ZP value outside the range from −30 to 30 mV exhibits good suspension stability [59,60].

On the note, the positively charged nanocarriers could bind with negatively charged mucosal membrane ideally, and thus facilitating the enhanced delivery of MMC drug and cellular uptake [61,62].

### 3.2. Reaction Yield, Drug Loading Capacity, and Encapsulation Efficiency

Table 4 provides information about the drug loading capacity (DLC), encapsulation efficiency (EE), and reaction yield of the formulated drug nanocarriers when different concentrations of encapsulated MMC drug (in the range of 0.25–1.50 mg/mL) were used. The highest DLC and EE were achieved to be 44.52 ± 1.05% and 60.31 ± 0.49% with 1.0 mg/mL concentration of MMC. Likewise, the DLC and EE were seen to reach saturation limit at 1.00 mg/mL of TPP, where the reduction in DLC and EE was observed inversely proportional to the increased concentration of TPP. Such limitation in DLC and EE suggests that the formulation having the particle size of 175 nm (as mentioned in Section 3.1.6) can hold approximately 44.52 ± 1.05% at its maximum value. Since the DLC and EE for both MMC with concentrations of 0.50 and 1.00 mg/mL show no significant differences, and thus the MMC with 0.5 mg/mL was chosen for further drug release studies.

### 3.3. Drug Release Kinetics Based on Different Types of Pharmacokinetics Models

**Figure 7:**
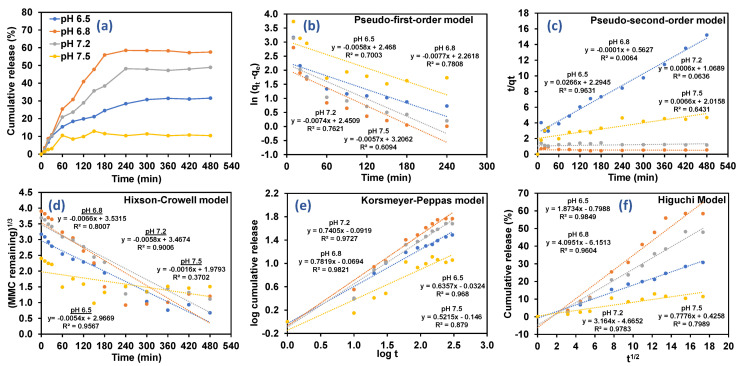
Drug release in (**a**) different release medium with pH 6.5, 6.8, 7.2 and pH 7.5; and drug release data fitted with five different pharmacokinetics models (**b**) Pseudo-first-order model, (**c**) Pseudo-second-order model, (**d**) Hixson-Crowell model, (**e**) Korsmeyer-Peppas model, and (**f**) Higuchi model.

In vitro release study of MMC was carried out in four different release mediums (phosphate buffer solution, PBS) with pH 6.5, 6.8, 7.2, and 7.5 to evaluate the drug release profile ranging from 0 to 480 min as a function of time. The release of loaded drugs from nanocomposites in different pH was conducted to study the mechanisms occurring inside the nanomatrix and to understand the pH response. From the graph in Figure 7a, it is clearly shown that the MMC release in release medium is highest in pH 6.8 with cumulative release of 56.48% followed by cumulative release of 50.22%, 30.88%, and 10.75% in the release mediums having the pH of 7.2, 6.5, and 7.5, respectively. This study validates that the amino group (−NH_2_) of CS was successfully protonated in slightly acidic condition [53,54], and the breakage of amino group attached at the surface of naocomposite then accelerates a greater amount of drug to get diffused out from the CS matrix. This phenomenon hence facilitates the drug release prior to the swelling behavior of CS NPs in various pH mediums. At higher pH, the swelling is quite limited and drug release is slightly slow. As reported in a similar study, the observation of sustained release for the same pH (up to 720 min) might be due to the interaction of amino group of CS with that of the carboxyl group of MMC [63].

Briefly, the drug release profile can be subdivided into two major phases, first phase or burst release phase, followed by second phase which involves sustain release phase [64]. In the first phase, the burst effect might be contributed to by the adsorption and attachment of drugs on the surface of nanocomposites with poor interaction, which can be clearly seen during the first 180 min [48]. Meanwhile, the second phase remains plateaued within the time from 180 min up to 480 min, which validates the sustained release drug from the core compartment of CS-Mn:ZnS nanocomposite [52]. This finding was strongly supported by previous work, which shows the burst release of MMC for the first 4 h (180 min) with sustained release up to 72 h.

In this study, five pharmacokinetic models were implemented to evaluate the drug release data and associated drug release mechanism. Five pharmacokinetics models including pseudo-first-order, pseudo-second-order, Hixson–Crowell, Korsmeyer–Peppas and Higuchi were used to fit the experimental release as illustrated in Figure 7b–f, respectively. Acquired fitting parameters such as K_1_, K_2_, K_hc,_ N, K_KP_, K_H_ as well as the correlation coefficient for each kinetic model were successfully tabulated in Table 5.

In pseudo-first-order kinetics model, the *q_e_* and *q_t_* represent the amount of MMC release at equilibrium and at certain time, respectively, and *K*_1_ represents the reaction coefficient, and *t* represent time [65].

(10) $$\;\;\;\;\;\;\ln\left(q_{e}-q_{t}\right)=\ln q_{e}-K_{1}t\;$$

In pseudo-second-order kinetics model, *K*_2_ represents the rate constant in pseudo-second-order model.

(11) $$\frac{t}{q_{t}}=\frac{1}{K_{2}}q_{e}^{2}+\frac{t}{q_{e}}$$

In addition, Hixson–Crowell reveals the relationship between the cube root of MMC remaining in nanocarriers as a function of time, where *K_HC_* is the rate constant, and *M*_0_ is the initial concentration of drug in the nanoparticles, for Hixson–Crowell model.

(12) $$\sqrt[{3}]{\left(M_{0}-q_{t}\right)}=K_{HC}t$$

For spherical particles with a granular matrix containing a water soluble drug, the release kinetics may be described using Kosmeyer–Peppas or Higuchi models [57,58]. The data for first 60% of drug release fraction (*q_t_*) were fitted with both models to investigate the drug release mechanisms. The drug release kinetic parameters for MMC-CS-Mn:ZnS nanocarriers were calculated using linearized form of Korsmeyer–Peppas and Higuchi models [66] as presented in Equations (13) and (14), respectively.

In the Higuchi model, the correlation between the amounts of MMC released was studied against ascending square root of time, where, K_H_ is denoted as the rate constant for Higuchi model.

(13) $$q_{t}=K_{H}\sqrt{t}$$

In the Korsmeyer–Peppas model, the relationship of log of the MMC released was studied versus the log of time, where log *q_t_* denotes the fraction released by time *t* (min), *n* is an exponent related to the drug release mechanism, *k* (*h*−*n*) is a rate constant.

(14) $$\log q_{t}{}_{\;}=n\log t+\log k$$

From the kinetics and mathematical models, it is clearly demonstrated that the release of MMC was proficient in CS-Mn:ZnS nanocarriers, fitting both the Higuchi model and Korsmeyer-Peppas model well. Hence, overall results manifested a more significant prospective upon the diffusion controlled mechanism [67] which involved the correlation of cumulative drug release proportionally with the function square root of time at equilibrium, with the correlation coefficient values (R^2^) of 0.9849, 0.9604, and 0.9783 for drug release in pH 6.5, 6.8 and 7.2, respectively. Separately, further validation on diffusion controlled mechanism was obtained with the R^2^ value of 0.8790 for pH 7.5, further fit using the Korsmeyer–Peppas model. From this model, we can determine the release exponent (*n*). The *n* values were found to be 0.63, 0.78, 0.74, and 0.52 for pH 6.5, 6.8, 7.2, and 7.8, respectively. The mechanism of drug release may be detailed by the adoption of initial 60% of the semi-empirical model and is known as the Korsmeyer–Peppas model. The value *n* in the model reflects the possible release mechanisms of the drug. The value of *n* < 0.5 indicates Fickian diffusion, whereas *n* > 0.5 indicates anomalous diffusion [68], as mentioned in Table 6.

Moreover, in the Korsmeyer–Peppas release model (the second-highest correlation coefficient values), the release exponent, *n*, obtained between 0.45 ≤ *n* ≤ 0.89 represents non-Fickian (anomalous) transport which involves hybrid drug release mechanism diffusion in hydrated matrix and polymer relaxation [69]. The highest *n* value was observed in release medium pH 6.8 (*n* = 0.78) validating that the swelling of CS that was controlled by water diffusion mechanism, hence facilitating an efficient drug release process from the MMC@CS-Mn:ZnS nanocomposite [24].

## 4. Conclusions

In conclusion, the present study investigated the release profiles of MMC@CS-Mn:ZnS nanocomposite as an effective DDS for non-muscle invasive bladder cancer. The developed DDS when we applied the MMC concentration of 1.0 mg/mL, the DLC and EE was achieved to be 44.52 ± 1.80% and 60.31 ± 0.49%, respectively. Furthermore, for the drug releasing profile conducted in PBS at four different solutions having the pH of 6.5, 6.8, 7.2 and 7.5, we observed the highest cumulative drug release of 56.48% in pH 6.8 media followed by 50.22%, 30.88%, and 10.75% release in the mediums having the pH of 7.2, 6.5, and 7.5, respectively. Additionally, the drug release data was fitted using five different pharmacokinetic models where the cumulative MMC release suits the Higuchi model well, revealing the diffusion-controlled mechanism which involved the correlation of cumulative drug release proportional to the function square root of time at equilibrium. In addition, the drug release studies with the correlation coefficient values (R^2^) of 0.9849, 0.9604, 0.9783, and 0.7989 for the pH of 6.5, 6.8, 7.2, and 7.5, respectively, were observed. Based on the overall results, we observed high drug loading capacity for the developed DDS, which is one of the key features to serve the MMC@CS-Mn:ZnS nanocomposite as an excellent drug nanocarrier system that eventually contributes to the improved chemotherapeutic efficiency.

## Figures and Tables

**Table 1:** Absorption peaks for different functional groups in NPs.

Wavenumber (nm^−1^)	Functional Group
3300	C–H and NH_2_ stretching in CS and MMC
2110	C–O stretching in CS
1638	NH_2_ bending in CS and MMC
1530 and 1402	C–O stretching of carboxylic group
1110, 655 and 610	Zn–S vibration

**Table 2:** Variation of different structural parameters of different types of NPs.

Sample	*hkl*	2θ (deg.)	β (rad.)	D (nm)	$\delta \times 10^{1}$	$\varepsilon \times 10^{2}$	*d_hkl_* (nm)	*a* (nm)
ZnS	[111]	28.69	0.0805	1.775	3.172	1.951	0.311	0.733
Mn:ZnS	[111]	28.70	0.0951	1.503	4.421	2.300	0.311	0.734
CS-Mn:ZnS	[111]	28.22	0.1117	1.279	6.111	2.709	0.316	0.740
MMC@CS-Mn:ZnS	[111]	28.75	0.0749	1.909	2.741	1.814	0.310	0.733

**Table 3:** Evaluation of particle sizes, PDI and zeta potential for NPs.

Sample	Particle Sizes (nm)	PDI	Zeta Potential (mV)
Mn:ZnS	46 ± 0.56	0.564	−22.80 ± 0.33
CS NPs	158 ± 0.21	0.289	+32.60 ± 0.29
CS-Mn:ZnS	161 ± 0.67	0.320	+32.70 ± 0.46
MMC@CS-Mn:ZnS	175 ± 0.33	0.448	+33.20 ± 0.38

**Table 4:** Data of DLC, EE, and reaction yield for nanocarriers with different encapsulation with MMC of varying concentration was used.

Nanocarriers	Drug Loading Capacity (%)	Encapsulation Efficiency (%)	Reaction Yield (%)
MMC@CS-Mn:ZnS ^a^	35.55 ± 1.25	50.13 ± 0.54	50.75
MMC@CS-Mn:ZnS ^b^	42.36 ± 1.80	60.01 ± 0.28	54.17
MMC@CS-Mn:ZnS ^c^	44.52 ± 1.05	60.31 ± 0.49	53.30
MMC@CS-Mn:ZnS ^d^	37.82 ± 1.45	55.28 ± 0.31	52.29

Notes: ^a^ 0.25 mg/mL, ^b^ 0.50 mg/mL, ^c^ 1.00 mg/mL, ^d^ 1.50 mg/mL of MMC.

**Table 5:** Kinetic parameter for drug release from CS-Mn:ZnS nanocomposite fitted to various pharmacokinetics models.

Model	Pseudo-First Order		Pseudo-Second Order		Hixson–Crowell		Korsmeyer–Peppas			Higuchi	
Release medium	K_1_	R^2^	K_2_	R^2^	K_HC_	R^2^	*n*	K_KP_	R^2^	K_H_	R^2^
pH 6.5	0.0058	0.7003	0.0266	0.9631	0.0054	0.9567	0.63	0.0324	0.9680	1.8734	0.9849
pH 6.8	0.0077	0.7808	0.0001	0.0064	0.0066	0.8007	0.78	0.0694	0.9821	4.0951	0.9604
pH 7.2	0.0074	0.7621	0.0006	0.0636	0.0058	0.9006	0.74	0.0919	0.9727	3.1640	0.9783
pH 7.5	0.0057	0.6094	0.0066	0.6431	0.0016	0.3702	0.52	0.1460	0.8790	0.7776	0.7989

**Table 6:** Variability of release exponent (*n*) and the respective release mechanisms.

Release Exponent (*n*)	Drug Transport Mechanism	Rate as a Function of Time (t) Transformation
*n* ≤ 0.45	Fickian diffusion	t^−0.5^
0.45 ≤ *n* ≤ 0.89	Non-Fickian transport	t*^n^*^−1^
*n* ≥ 0.89	Case II transport	t
*n* ≥ 1	Super case II transport	t*^n^*^−1^

## Data Availability

Data can be provided upon request.
